# Heterotrophically Ultrahigh-Cell-Density Cultivation of a High Protein-Yielding Unicellular Alga *Chlorella* With a Novel Nitrogen-Supply Strategy

**DOI:** 10.3389/fbioe.2021.774854

**Published:** 2021-11-22

**Authors:** Quan Xu, Guoli Hou, Jianping Chen, Hongxia Wang, Li Yuan, Danxiang Han, Qiang Hu, Hu Jin

**Affiliations:** ^1^ Center for Microalgal Biotechnology and Biofuels, Institute of Hydrobiology, Chinese Academy of Sciences, Wuhan, China; ^2^ College of Advanced Agricultural Sciences, University of Chinese Academy of Sciences, Beijing, China; ^3^ Key Laboratory for Algal Biology, Institute of Hydrobiology, Chinese Academy of Sciences, Wuhan, China; ^4^ The Innovative Academy of Seed Design, Chinese Academy of Sciences, Beijing, China; ^5^ Institute for Advanced Study, Shenzhen University, Shenzhen, China; ^6^ State Key Laboratory of Freshwater Ecology and Biotechnology, Institute of Hydrobiology, Chinese Academy of Sciences, Wuhan, China

**Keywords:** *Chlorella*, heterotrophic cultivation, high-cell-density, protein, nitrogen supply

## Abstract

The unicellular green alga *Chlorella* is an ideal protein source. However, the high production cost and low production capability of the current main photoautotrophic culture mode limit its application especially as an alternative protein source for food and feed, which might be overcome through high-cell-density cultivation in fermenters. In this study, a *Chlorella sorokiniana* strain CMBB276 with high protein content was selected from five *Chlorella* strains by comprehensive evaluation of their growth rates, protein contents, and yields. The optimal cultural temperature, pH, and mole ratio of carbon and nitrogen (C/N) for *C. sorokiniana* CMBB276 growth were found to be 30°C, 6.5, and 18, respectively. Ammonium chloride was proved to be the best nitrogen (N) source for *C. sorokiniana* CMBB276 growth, whereas growth inhibition caused by the accumulation of salts was observed under fed-batch cultivation when maintaining a constant C/N ratio of 18 by controlling pH with sodium hydroxide solution. By simultaneously reducing the concentration of ammonium chloride in the feeding medium and controlling pH with ammonium hydroxide, we finally achieved the ultrahigh-cell-density cultivation of *C. sorokiniana* CMBB276. The highest biomass concentration and protein yield reached 232 and 86.55 g l^−1^, respectively, showing the great potential of culturing *C. sorokiniana* CMBB276 in fermenters for economic and large-scale protein source production.

## Introduction

A growing global population and a higher demand for more and better quality food will place increased pressure on the world’s natural resources (Henchion et al., 2017). Protein is an essential nutritional component in the human diet, providing most of the nitrogen humans need. The global protein demand is approximately 202 million tons, and it was estimated that the world demand for animal-derived protein will double by 2050, resulting in concerns for sustainability and food security (Henchion et al., 2017). Microalgae are able to synthesize all amino acids, and the average protein content of most microalgae is equal to or even higher than conventional plant protein sources (Madeira et al., 2017). Therefore, microalgae are considered one of the most promising feedstocks for sustainable supply of commodities for both food and non-food products, and developing microalgae for feed and food is a viable strategy that can potentially contribute to global food security (Matsuda et al., 2011; Madeira et al., 2017; Torres-Tiji et al., 2020).

The unicellular green alga *Chlorella* is one of the few microalgae largely employed for human consumption, which has high protein content with a balanced amino acid composition (Becker, 2007; Liu and Hu, 2013). A large number of nutritional and toxicological evaluations have also demonstrated the suitability of *Chlorella* biomass as a valuable feed supplement or substitute for conventional protein sources (e.g., soybean meal, fish meal, and rice bran) (Madeira et al., 2017). However, commercial production of *Chlorella* biomass used in healthy foods and feeds of animals is provided mostly in open raceway ponds and this commonly used way of algal cultivation is characterized by rather low production rates and high production cost, limiting the widespread application of *Chlorella* as a food or feed commodity (Liu and Hu, 2013).

Heterotrophic cultivation of some microalgae (e.g., *Chlorella*) in fermenters may provide a cost-effective, large-scale alternative. The microalgal biomass yields from heterotrophic systems can be 50 or 100 times greater than those of photo-autotrophic systems (Stephenson et al., 2010; Davis et al., 2011). Protein content, one of the most important nutritional characteristics, is directly related to the quality of microalgal biomass (Kovač et al., 2013). Thus, the achieved biomass concentration/productivity, together with the algal quality (protein content), will determine the final market value and application of heterotrophic *Chlorella* biomass as a protein source in the food and feed fields. However, to date, the reported maximum biomass concentration/productivity of most microalgae under heterotrophic culture is still not as competitive as that of the other industrial microorganisms (e.g., bacteria or yeast) (Jin et al., 2020). Therefore, further reduction in the production cost of *Chlorella* biomass relies greatly on the improvement of biomass concentration/productivity.

In the present study, a promising industrial algal *C. sorokiniana* CMBB276 for protein production was screened out by comparing its protein content/yield with other four protein-producing *Chlorella* strains in flask cultivation*.* Then, the optimal heterotrophic growth condition of *C. sorokiniana* CMBB276 was established by optimizing important nutritional and environmental factors (e.g., pH, temperature, and C/N ratios) through batch fermenter cultivation. By using ammonium chloride as N source, gradual accumulation of salts and growth inhibition were observed when maintaining a constant C/N ratio and controlling pH with sodium hydroxide solution under fed-batch cultivation in fermenters. We finally achieved the ultrahigh-cell-density cultivation of *C. sorokiniana* CMBB276 by simultaneously reducing the concentration of ammonium chloride in the feeding medium and controlling pH with ammonium hydroxide instead of sodium hydroxide solution.

## Materials and methods

### Strains and culture conditions

Five freshwater protein-yielding *Chlorella* strains (designated as *C.* sp. CMBB266, *C. sorokiniana* CMBB273, *C.* sp. CMBB152, *C. sorokiniana* CMBB276, and *C.* sp. CMBB277) capable of heterotrophic growth were provided by Prof. Guoxiang Liu from the Institute of Hydrobiology, Chinese Academy of Sciences. These algal cells were maintained in a modified Endo growth medium (Jin et al., 2020), containing glucose 30 g l^−1^, KNO_3_ 3 g l^−1^, KH_2_PO_4_ 1.2 g l^−1^, MgSO_4_•7H_2_O 1.2 g l^−1^, trisodium citrate 0.2 g l^−1^, FeSO_4_•7H_2_O 0.016 g l^−1^, EDTA-Na_2_ 2.1 mg l^−1^, CaCl_2_•2H_2_O 0.105 g l^−1^, H_3_BO_3_ 2.86 mg l^−1^, ZnSO_4_•7H_2_O 0.222 mg l^−1^, MnCl_2_•4H_2_O 1.81 mg l^−1^, Na_2_MoO_4_•2H_2_O 0.021 mg l^−1^, and CuSO_4_•5H_2_O 0.07 mg l^−1^. The initial pH of the modified Endo medium was adjusted to 6.5 with 3 M NaOH solution.

For the screening experiment, single colonies of the above five *Chlorella* strains were first inoculated into 100-ml Erlenmeyer flasks containing 20 ml of sterile modified Endo medium in an orbital shaker (180 rpm, 30°C) in the darkness for 5–6 days. The algal cells were then inoculated into 250-ml Erlenmeyer flasks containing 100 ml modified Endo medium with an inoculum volume of 1% (v v^−1^) and grown with the same culture conditions for 6 days.

Sequential optimizations of temperature, pH, C/N ratio, and types of N source were conducted in 1-l bioreactors (Applikon Biotechnology, Delft, Netherlands) with the working volume of 450 ml by inoculating 10% (v v^−1^) of the exponential growing algal cells in flask culture. Different cultural temperatures (25°C, 28°C, 30°C, and 32°C) were maintained to investigate their effects on *C. sorokiniana* CMBB276 growth. Subsequently, at the optimal growth temperature of 30°C, the different pH values (5.0, 5.5, 6.0, 6.5, 7.0, and 7.5) were maintained during the whole fermentation. To investigate the effects of different C/N ratios on the biomass growth, the initial glucose was fixed at 30 g l^−1^, while the concentration of KNO_3_ was changed to maintain the mol C/N ratios at different values (6, 12, 18, and 24) under the optimal temperature (30°C) and pH (6.0). To obtain the optimal N source for *C. sorokiniana* CMBB276 growth, potassium nitrate (KNO_3_) in the above modified Endo medium was replaced by other N sources with the same concentration of nitrogen (corresponding to 0.7 g N l^−1^). The agitation speed and air flow rate for the above optimization experiments in 1-l bioreactors were controlled at 200 rpm and 500 ml min^−1^, respectively.

The fed-batch culture experiments were conducted in a 7.5-l fermenter (BioFlo and CelliGen 310, Eppendorf, Framingham, MA, USA) initially containing 2.8 l of the optimized growth medium (30 g l^−1^ glucose, 2.71 g l^−1^ ammonium chloride, 1.2 g l^−1^ KH_2_PO_4_, 1.2 g l^−1^ MgSO_4_•7H_2_O, 0.2 g l^−1^ trisodium citrate, 0.016 g l^−1^ FeSO_4_•7H_2_O, 2.1 mg l^−1^ EDTANa_2_, 0.03 g l^−1^ CaCl_2_•2H_2_O, 2.86 mg l^−1^ H_3_BO_3_, 0.222 mg l^−1^ ZnSO_4_•7H_2_O, 1.81 mg l^−1^ MnCl_2_•4H_2_O, 0.021 mg l^−1^ Na_2_MoO_4_, 0.07 mg l^−1^ CuSO_4_•5H_2_O). The optimized culture conditions (pH 6.5, 30°C, and mol C/N ratio 18) were directly applied to the culture process in the 7.5-l fermenter. The pH was maintained at 6.5 ± 0.1 by automatic addition of 3 M NaOH solution. The aeration rate was maintained at 1 vvm with the initial airflow rate of 2.8 l min ^−1^. The dissolved oxygen (DO) was controlled at about 20% by coupling it with the agitation speed. The glucose concentrations were controlled in the range of 5–10 g l^−1^ with the stepwise constant feeding strategy proposed previously (Jin et al., 2020). The feeding medium was the 25-fold concentrated growth medium. To ensure the reliability of the results, all the above experiments were repeated for three times.

### Analytical procedures

Cell growth was monitored by measuring the dry biomass weight as reported previously (Jin et al., 2020). Briefly, 1 ml of the cell cultures was sampled to determine the biomass concentration. Cells were washed with distilled water before being filtered on a pre-weighted Whatman GF/C filter (Whatman, Buckinghamshire, UK). The filters containing *Chlorella* were dried in an oven at 105°C for 24 h, then taken out and cooled to room temperature in a vacuum dryer and weighed. The weight of *Chlorella* cells was calculated and expressed in grams per liter as the biomass concentration.

The protein content of *Chlorella* was determined with a modified Kjeldahl method (Jung et al., 2003). The total nitrogen content in the freeze-dried algal powder was determined by the automated Kjeldahl analyzer (UDK159-VELP, Italy), and the protein content of *Chlorella* was calculated with the conversion factor of 6.25. Briefly, samples of 0.1–0.2 g were accurately weighed out and then digested with 10 ml of concentrated sulfuric acid in the presence of a catalyst by using a digestion system (DK20, Italy). The catalyst is a mixture of potassium sulfate (2.8 g), copper sulfate pentahydrate (0.08 g), and titanium dioxide (0.08 g). Forty percent of NaOH (w/v) was used to produce an alkaline distillation environment, and 4% boric acid (w/v) solution was used to collect ammonia from distilled water. The titrations were performed with standardized 0.1 N hydrochloric acid. The mixed indicator regent (0.1 g of methyl red and 0.1 g of bromocresol green in 100 ml of 95% ethanol) was used to identify the end point of the titration.

The glucose concentration was determined with a Safe-Accu UG Blood Glucose Monitoring System (model BGMS-1, Sinocare Inc., Changsha, China). The Na^+^ concentration of fermentation broth was determined using the Biochemical Analyzer (BioProfile 300A, Nova, Waltham MA, USA) after centrifugation and filtration through a 0.22-μm filter membrane (Millipore, USA).

### Statistical analysis

The values were expressed as mean ± standard deviation. The data were analyzed by one-way ANOVA using SPSS (version 19.0). A statistically significant difference was considered at *p* < 0.05.

## Results

### Screening of the *Chlorella* Strain With the Potential in Producing Proteins Under Heterotrophic Culturing Conditions

**FIGURE 1:**
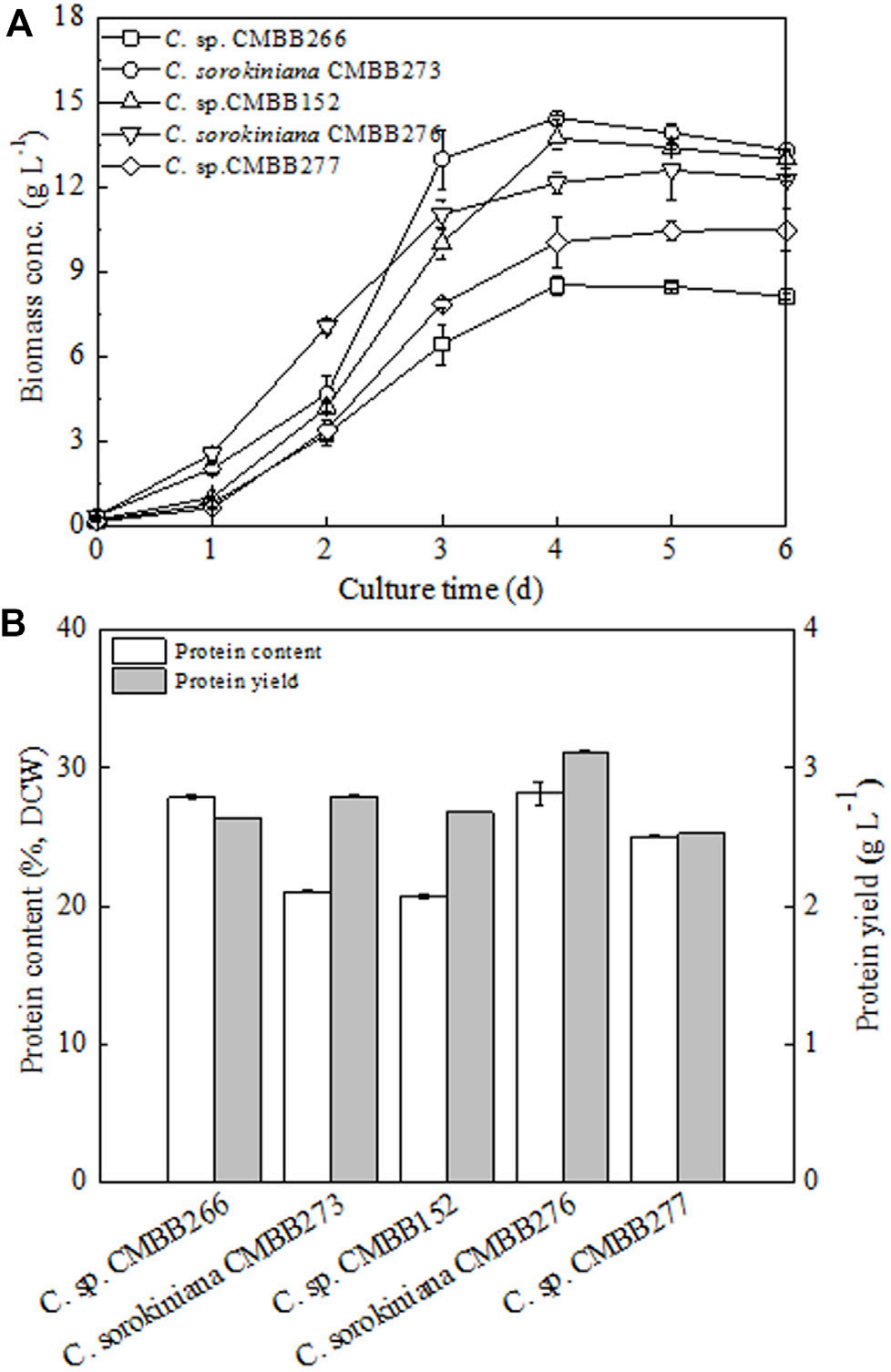
Comparison of cellular growth rates **(A)**, protein contents, and yields **(B)** of five *Chlorella* trains under heterotrophic culture. Cells on day 6 were harvested for protein analysis. Values are shown as mean ± SD from three biological independent replicates.

To obtain the optimal *Chlorella* stain suitable for protein production, a comprehensive evaluation of both the cellular growth and protein production performances of five *Chlorella* strains were conducted through primary flask cultivation. Under heterotrophic cultivation in flasks, three *Chlorella* strains (CMBB273, CMBB152, and CMBB276) exhibited a faster cellular growth rate and higher maximum biomass concentration compared to the other two strains (CMBB277 and CMBB266) (Figure 1A). The final biomass concentration of the fastest-growing alga CMBB273 reached 13.22 g l^−1^, which was 40.0% higher than that of the slowest-growing alga CMBB266 (9.47 g l^−1^) (*p* < 0.05). In contrast, the cellular protein content of CMBB266 (27.8%) was 32% higher than that of CMBB273 (21.0%) (*p* < 0.05). Consequently, a similar final protein yield (2.6–2.7 g l^−1^) was observed for these two strains (Figure 1B). Among the five *Chlorella* strains, the highest protein yield (3.48 g l^−1^) was observed for *C. sorokiniana* CMBB276 due to its highest cellular protein content (28.1%) and a relatively high biomass concentration (12.47 g l^−1^). Thus, *C. sorokiniana* CMBB276 was chosen as the best protein-yielding strain for the subsequent investigation.

### Optimization of Heterotrophic Culturing Conditions for *C. sorokiniana* CMBB276

**FIGURE 2:**
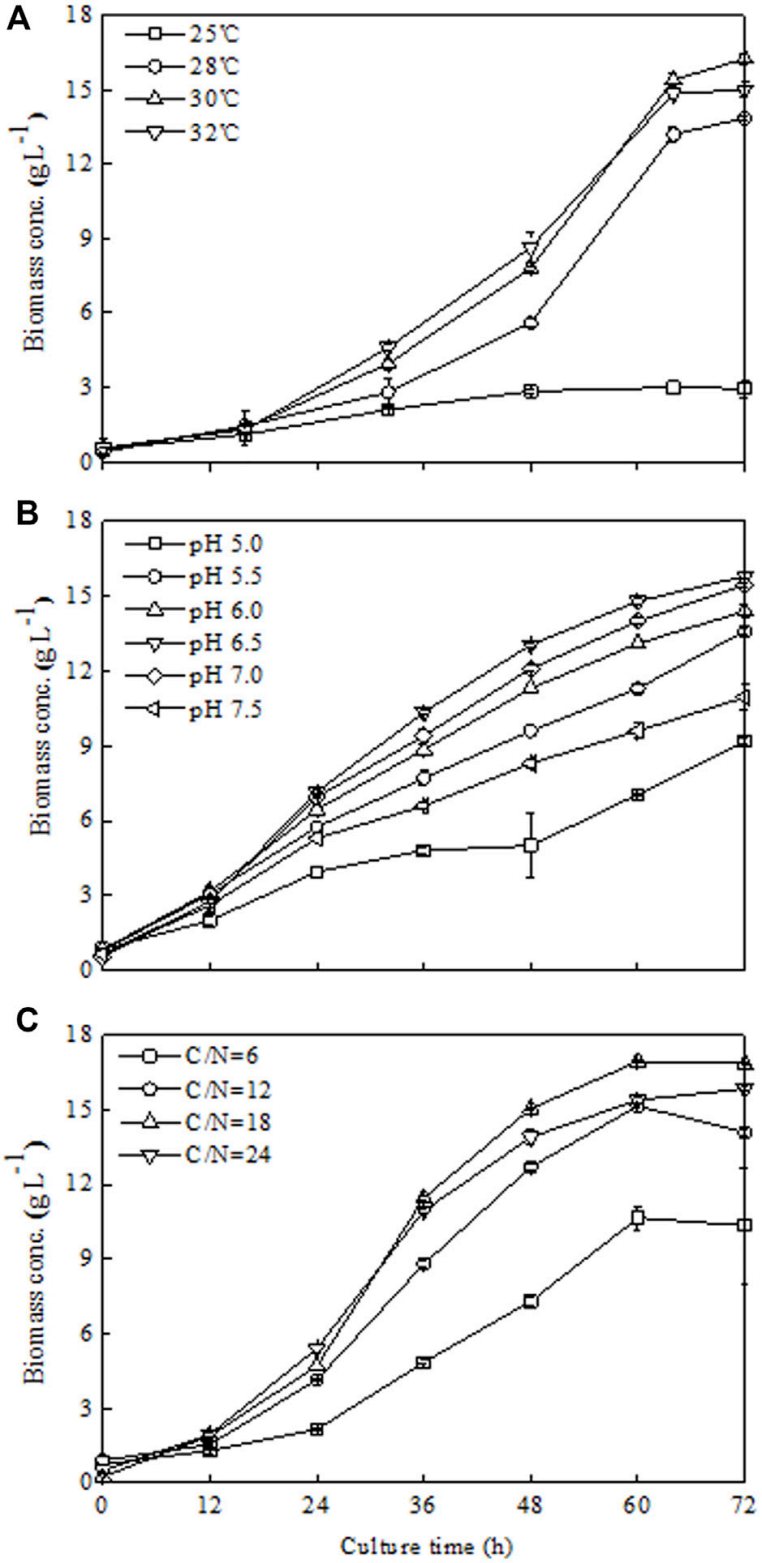
Effects of different temperatures **(A)**, pH **(B),** and C/N ratios **(C)** on *C. sorokiniana* CMBB276 growth under heterotrophic conditions in 1-l bioreactors. Values are shown as mean ± SD from three biological independent replicates.

The effect of temperature on *Chlorella sorokiniana* CMBB276 growth was investigated in 1-l bioreactors. As indicated in Figure 2A, *C. sorokiniana* CMBB276 grew slowest when cultured at 25°C, the biomass growth of *C. sorokiniana* CMBB276 nearly stopped after 48 h, and the highest biomass concentration was less than 3 g l^−1^. An obvious increase in growth rate could be observed when the culture temperatures were controlled in the range from 28°C to 32°C. During the whole culture period, the fastest cellular growth and highest biomass concentration (16.25 g l^−1^) could be obtained at 30°C. Thus, the following optimization experiments were conducted at 30°C.

As shown in Figure 2B, *C. sorokiniana* CMBB276 preferred to grow at a neutral or weak acidic environment (pH 6–7). The cellular growth of *C. sorokiniana* CMBB276 slowed down significantly when the culture pH was beyond this range (*p* < 0.05). The fastest cellular growth and highest biomass concentration could be observed when pH was maintained at 6.5. Therefore, 6.5 was chosen as the optimal pH for the following experiments.

Under the obtained optimal temperature (30°C) and pH 6.5, the effects of different mol C/N ratios (6, 12, 18, and 24) on the growth of *C. sorokiniana* CMBB276 were investigated. Among this C/N range, a low C/N ratio of 6 did not benefit to the growth of *C. sorokiniana* CMBB276, and both its cellular growth rate and maximum biomass concentration were the lowest (Figure 2C). It was found that culturing *C. sorokiniana* CMBB276 under the C/N ratio of 18 could achieve the fastest growth rate and the highest biomass concentration (16.85 g l^−1^) during the whole culture period (Figure 2C). Therefore, the subsequent experiments were conducted under a C/N ratio of 18.

**FIGURE 3:**
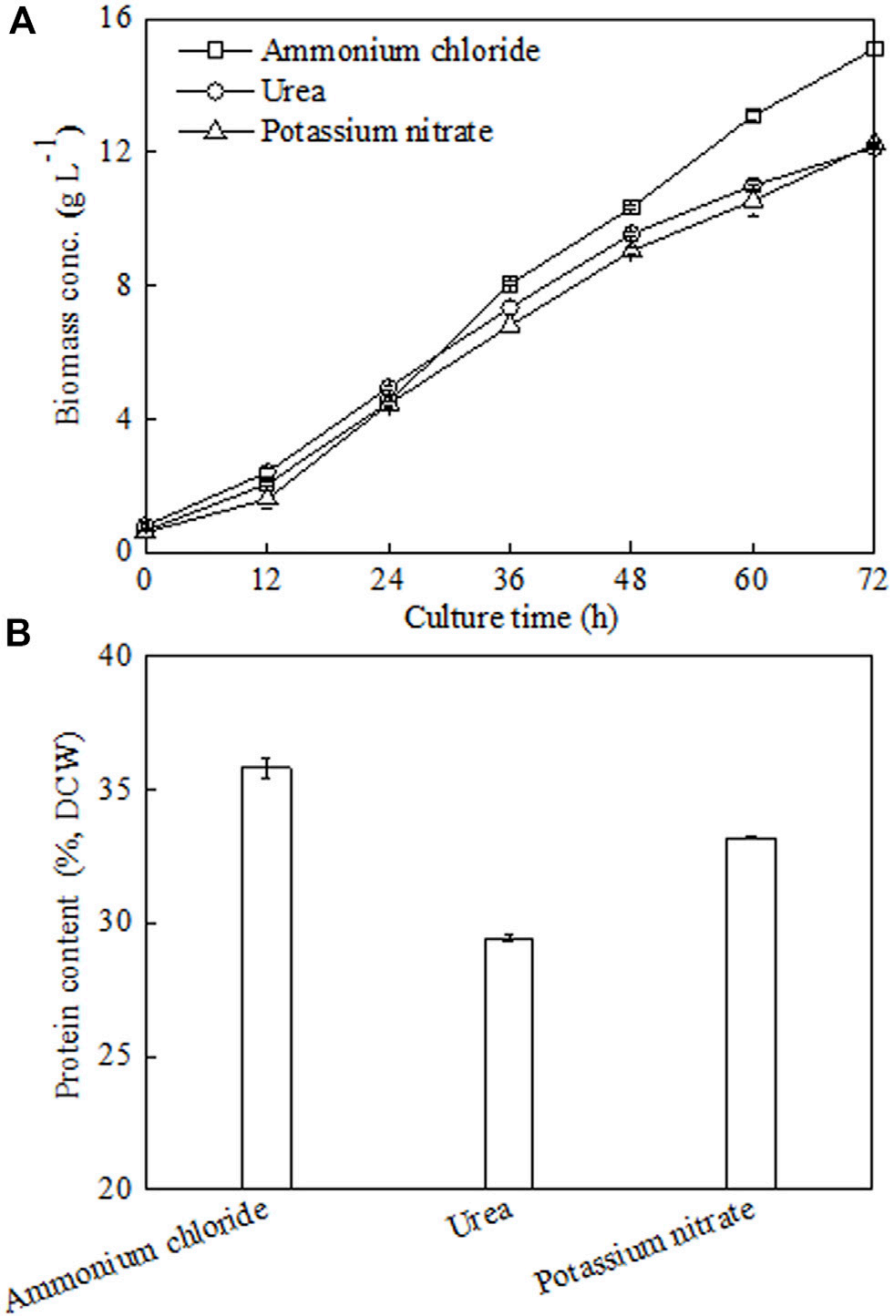
Effects of different nitrogen sources on *C. sorokiniana* CMBB276 growth **(A)** and protein content **(B)** under heterotrophic conditions in 1-l bioreactors. Values are shown as mean ± SD from three biological independent replicates.

Next, under batch fermenter cultivation, the effects of three different types of N sources (ammonium chloride, urea, and potassium nitrate) on *C. sorokiniana* CMBB276 growth and cellular protein content were investigated under the same C/N ratio of 18. Obviously, culturing *C. sorokiniana* CMBB276 with the N source of ammonium chloride favored both cellular growth and protein production, which achieved the maximum biomass of 15.1 g l^−1^ and the maximum protein content of 35.81% (Figure 3). Culturing *C. sorokiniana* CMBB276 with urea and potassium nitrate had similar cellular growth rates during the whole culture period (Figure 3A), but the lowest cellular protein content (29.44%) was observed when using urea as N source. Thus, ammonium chloride was chosen as the optimal nitrogen source for the subsequent experiments.

### Growth of *C. sorokiniana *CMBB276 Under Fed-Batch Cultivation

**FIGURE 4:**
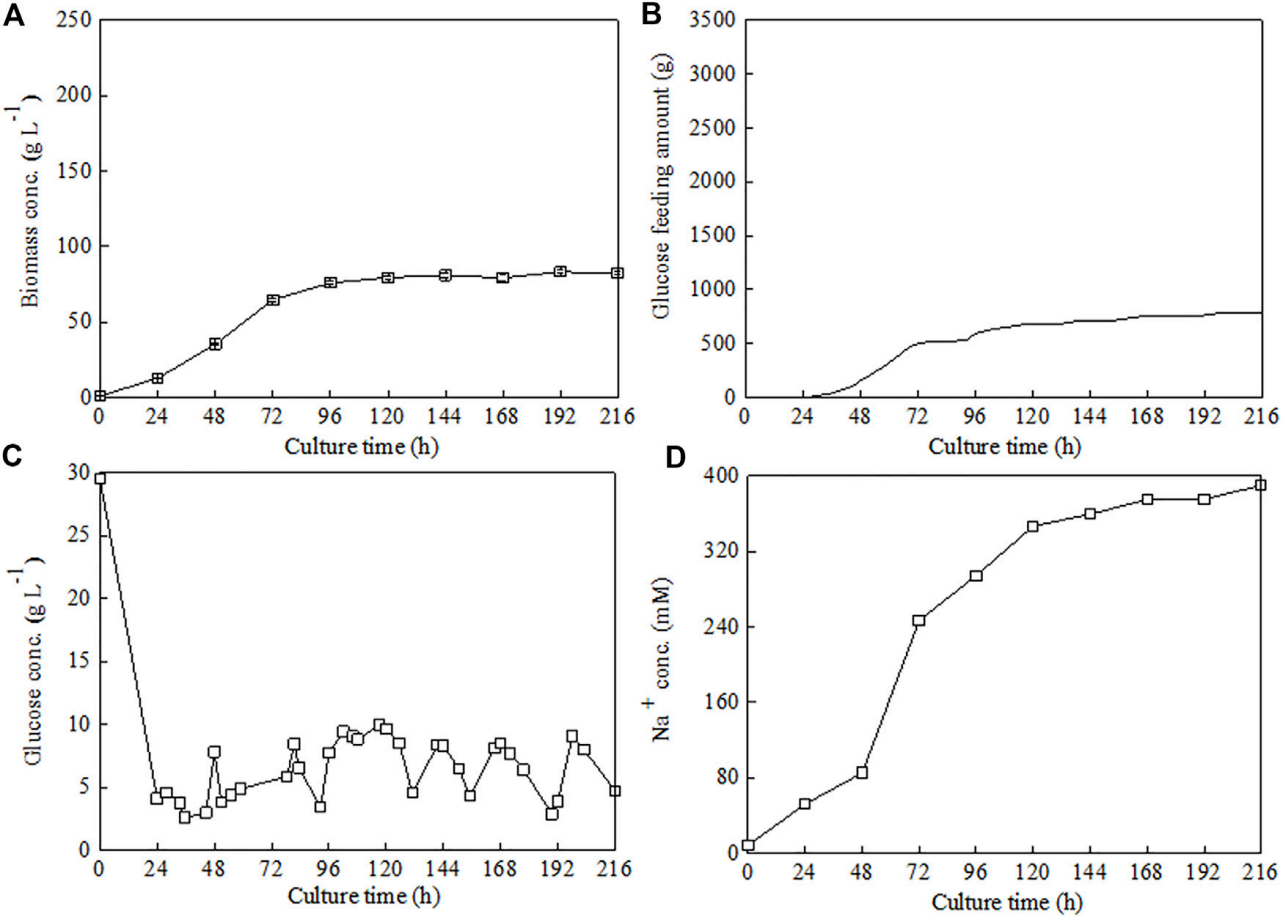
Time courses of *C. sorokiniana* CMBB276 growth **(A)**, glucose consumption **(B)**, glucose concentration **(C)**, and Na^+^ concentration **(D)** under fed-batch cultivation with ammonium chloride as nitrogen source in a 7.5-l fermenter. Sodium hydroxide solution was used for pH adjustment, and the C/N ratio of feeding medium was 18.

Under the above optimized culture condition (ammonium chloride as N source, C/N ratio 18, 30°C, and pH 6.5), high-cell-density fed-batch cultivation of *C. sorokiniana* CMBB276 was performed in 7.5-l fermenters with the previously proposed stepwise constant feeding strategy (Jin et al., 2020). After inoculation, glucose was quickly consumed from an initial 30 to 4.1 g l^−1^ during the first 24 h. Then, glucose feeding was started and the glucose concentrations during the whole feeding period were maintained in the range of 5–10 g l^−1^ (Figure 4C). Fast *C. sorokiniana* CMBB276 growth could be observed for the first 72 h, and the biomass concentration could reach a high level of 76 g l^−1^ (96 h). After this fast growth phase, the cellular growth rate decreased obviously and the maximum concentration was only 83.2 g l^−1^ during the whole culture period (Figure 4A), which could also be reflected from the change of glucose feeding amount (Figure 4B). It was found that to maintain a constant pH, a large amount of alkali solution (3 M NaOH) was consumed when using ammonium chloride as the N source, and the total consumption amount of NaOH solution reached as high as 400 ml (Table 1, Strategy 1#). During the exponential growth phase, the Na^+^ concentration increased quickly from 85.2 mM at 48 h to 246.8 mM at 72 h. The final Na^+^ concentration in the fermentation broth reached up to 389.4 mM (Figure 4D), which was mainly from the automatic addition of alkali solution (3 M NaOH) and was very close to the calculated Cl^−^ concentration (390.6 mM) from the total supply amount of nitrogen source (NH_4_Cl). It was speculated that the reduction in cellular growth rate after 72 h might be ascribed to the accumulation of salt (NaCl) caused by continuous alkali solution addition.

**TABLE 1:** Comparison of major production performance indices of *C. sorokiniana* CMBB276 in 7.5 L fermenters under different culture strategies.

Strategy	C/N ratio of feeding medium^a^	Alkali solution for pH maintenance	Alkali solution consumption amount (ml)	Final average C/N ratio	Maximum biomass conc. (g L^−1^) and protein content (%, DCW)	Maximum protein yield (g L^−1^)	Protein productivity (g L^−1^ d^−1^)	Conversion rate from glucose to protein (%,w/w)
1#	18:1	3 M NaOH	400 ± 25	18 ± 0.00	83.25 ± 0.75 (35.53 ± 0.14)	29.57 ± 0.38	3.70 ± 0.05	11.63 ± 0.15
2#	18:1	NH_3_·H_2_O	180 ± 11	5.95 ± 0.23	91.30 ± 0.20 (53.41 ± 0.05)	48.76 ± 0.15	7.50 ± 0.02	18.03 ± 0.05
3#	90:1	NH_3_·H_2_O	310 ± 17	8.47 ± 0.43	123.25 ± 3.85 (39.49 ± 0.11)	48.67 ± 1.66	8.11 ± 0.28	12.61 ± 0.43
4#	360:1	NH_3_·H_2_O	615 ± 12	14.02 ± 0.75	232.00 ± 1.00 (37.30 ± 0.25)	86.55 ± 0.95	9.62 ± 0.11	19.84 ± 0.22

a The glucose concentrations in the feeding medium were kept the same (750 g l^−1^) for different C/N ratios.

**FIGURE 5:**
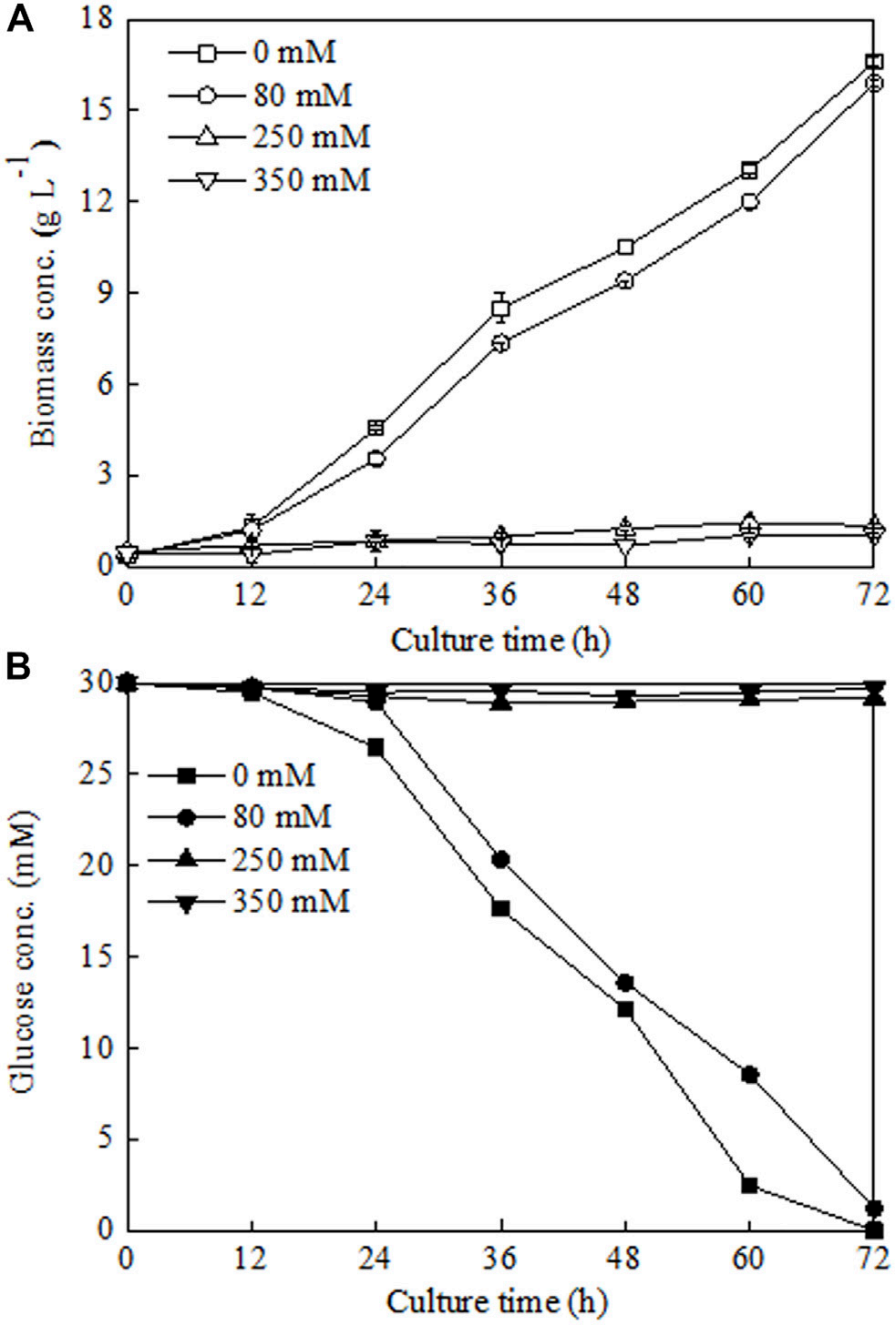
Comparison of biomass growth **(A)** and glucose consumption **(B)** under different sodium chloride addition amounts. Values are shown as mean ± SD from three biological independent replicates.

To verify this hypothesis, three different NaCl addition concentrations (80, 250, and 350 mM), which corresponded to three different Na^+^ concentrations observed in the middle exponential growth phase (48 h), late exponential growth phase (72 h), and stationary growth phase (144 h) (Figures 4A,D), were chosen to investigate their influences on *C. sorokiniana* CMBB276 growth in 1-l bioreactors. Compared to the control without NaCl addition, adding 80 mM NaCl had slight inhibition on *C. sorokiniana* CMBB276 growth, and the biomass concentrations for the same time were slightly lower than those of control (Figure 5A), which could also be reflected from the comparison of residual glucose concentration (Figure 5B). However, when exogenous NaCl addition amount reached above 250 mM, obvious growth inhibition could be observed, and both *C. sorokiniana* CMBB276 growth and glucose consumption nearly stopped under exogenous NaCl addition at 250 and 350 mM (Figure 5).

### Improvement of *C. sorokiniana *CMBB276 Growth With a Novel Nitrogen-Supply Strategy

**FIGURE 6:**
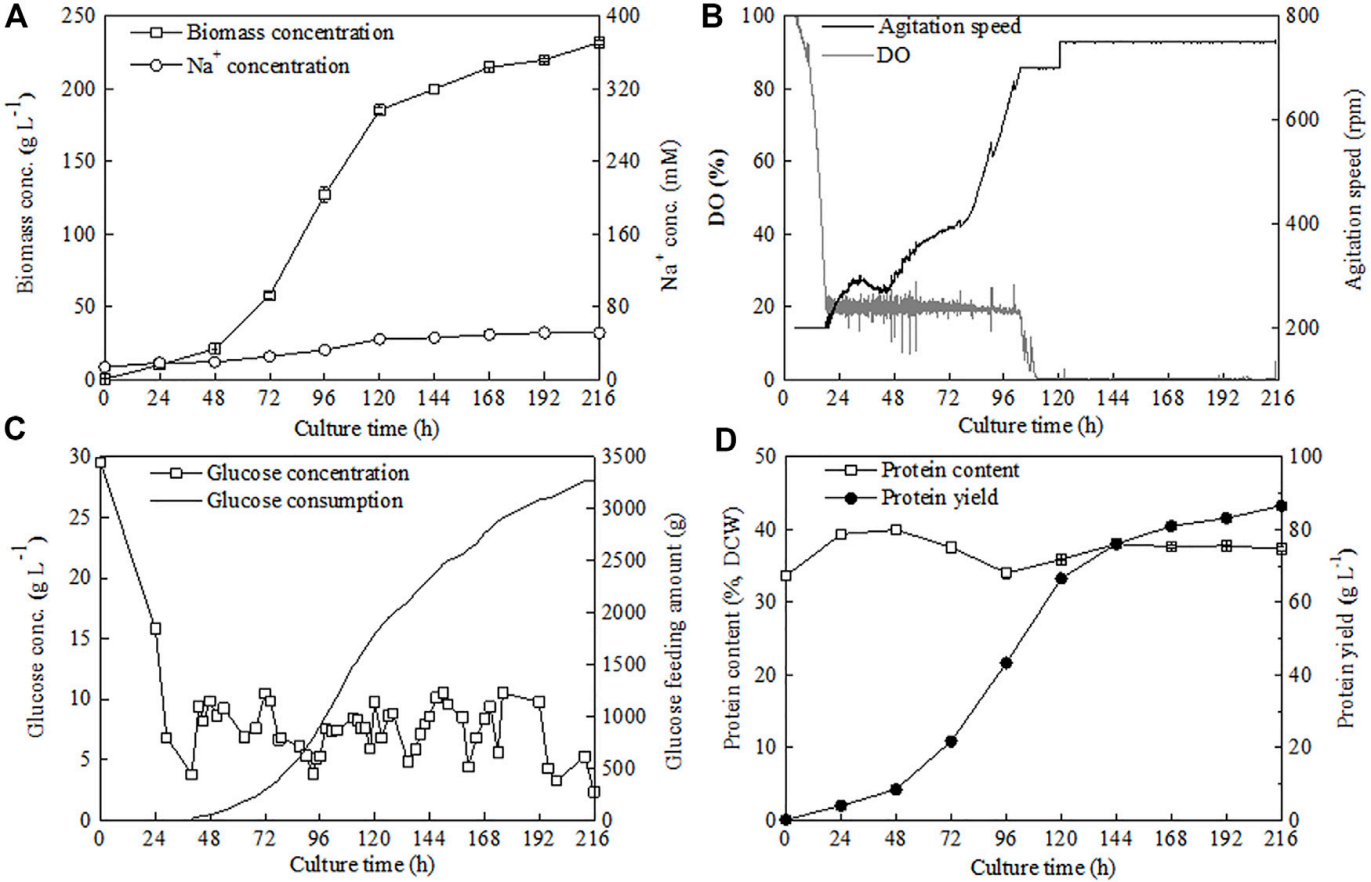
Time courses of biomass and Na^+^ concentrations **(A)**, DO and agitation speed **(B)**, glucose consumption and glucose concentration **(C)**, and protein content and protein yield **(D)** under novel N supply strategy by reducing the N concentration of feeding medium and adjusting pH with ammonium hydroxide.

To avoid the inhibitory effect of the generated salt (NaCl) caused by pH control on *C. sorokiniana* CMBB276 growth, the alkali solution (3 M NaOH) for pH adjusting was replaced with ammonium hydroxide (NH_3_•H_2_O, 25%, w/w). Meanwhile, considering the possible adverse effect of the low C/N ratio on cellular growth due to additional introduction of nitrogen nutrition by NH_3_•H_2_O addition, the NH_4_Cl concentration in the concentrated feeding medium was reduced to 1/20 of its former level (from 68 to 3.4 g l^−1^). By using this novel nitrogen supply strategy, the Na^+^ concentrations during the whole fermentation could be maintained below 80 mM and the maximum biomass concentration reached 232 g l^−1^ (Figure 6A), which was 2.8-fold of the former maximum concentration under a constant C/N ratio of 18 by controlling pH with NaOH solution. Under the preset maximum agitation speed of 750 rpm, it was hard to maintain DO constant by conventional air supply mode when the biomass concentration reached a high level close to 200 g l^−1^. Consequently, the cellular growth slowed down obviously after 120 h. However, continuous cellular growth could also be observed even though DO was maintained at 0 for a long culture period (Figures 6A,B). The final glucose feeding amount at 216 h with this novel nitrogen supply strategy reached 3271 g, which was 4.2-fold of the former strategy (Figures 4B, 6C). The protein content of *C. sorokiniana* CMBB276 during the whole fermentation was in the range of 33.6%–40.0% of dry cell weight (DCW), and the final protein content and maximum protein yield was 37.3% of DCW and 86.55 g l^−1^, respectively (Figure 6D; Table 1). It was observed that the C/N ratio in the feeding medium has great influence on the whole fermentation process when NH_3_•H_2_O was used for pH adjustment. The highest biomass concentration of 232 g l^−1^ could be achieved under a high feeding medium C/N ratio of 360 (low N concentration of 3.4 g l^−1^). Both the achieved maximum biomass concentration and the total ammonium hydroxide consumption amount reduced with the decline of the C/N ratio in the feeding medium (Table 1). Except for the adjustment of pH with 3 M NaOH solution (Strategy 1#), the final average C/N ratios for all other strategies were higher than the C/N ratios of the feeding medium due to the addition of ammonium hydroxide for pH maintenance. When the C/N ratio of the feeding medium was 360 (Strategy 4#), the calculated average C/N ratio for the whole fermentation was 14.02 (Table 1), which was close to the obtained optimal C/N ratio of 18 for *C. sorokiniana* CMBB276 growth under batch fermenter cultivation (Figure 2C).

## Discussion

The protein-rich *Chlorella* has long been used as a source of protein source and is produced mainly for human health food (Liu and Chen, 2016). The low production capacity and high production cost of the current commercial photoautotrophic cultivation mode limit the application of *Chlorella* biomass as bulk animal feed. The heterotrophic cultivation of *Chlorella* in fermenters is an effective pathway for economic production of *Chlorella* biomass. However, the economic advantages of heterotrophic cultivation over traditional photoautotrophic cultivation rely greatly on the achieved biomass concentration in fermenters (Jin et al., 2020). In this study, a high cell density culture process for the high protein-yielding *C. sorokiniana* CMBB276 was established through effective culture condition optimization. An ultrahigh biomass concentration (232 g l^−1^) and a record high protein yield (86.55 g l^−1^) were achieved with a novel nitrogen-supply strategy by adjusting pH with ammonium hydroxide replacing NaOH solution and simultaneously reducing the N concentration of feeding medium.

In general, microalgae are able to assimilate a variety of nitrogen sources, including ammonium (NH_4_^+^), nitrate (NO_3_^−^), and urea (Perez-Garcia et al., 2011). Ammonium is the most preferred and most energetically efficient nitrogen source for microalgal growth due to less energy requirement for its uptake and assimilation, which has been demonstrated for many *Chlorella* spp. and *Dunaliella* spp. (Perez-Garcia et al., 2011). When the culture pH was controlled, ammonium is a reliable nitrogen source (de-Bashan et al., 2005). However, *C. protothecoides*, *N. laevis*, and *P. tricornutum* exhibit a preference for nitrate or urea over ammonium (Shi et al., 2000; Wen and Chen, 2001; Lee and Lee, 2002). Our study indicated that among three common N sources (ammonium chloride, potassium nitrate, and urea), ammonium chloride was the optimal N source for *C. sorokiniana* CMBB276 growth when the same pH was maintained (Figure 3A). Besides cellular growth, it was found that the cellular protein content of *C. sorokiniana* CMBB276 was also affected by the type of nitrogen source. Under the same C/N ratio, higher protein content could be obtained when culturing *C. sorokiniana* CMBB276 with an N source of NH_4_^+^ (Figure 3B).

Maintaining a suitable C/N ratio and constant pH is very important for the high-cell-density cultivation of *Chlorella.* Usually, to achieve the fast growth, the optimal C/N ratio was kept for both initial batch medium and concentrated feeding medium (Jin et al., 2020). Meanwhile, to maintain the C/N ratio constant, sodium hydroxide (NaOH) and potassium hydroxide (KOH) are two common alkali used for pH adjustment (Xiong et al., 2008; Jin et al., 2021; Ma et al., 2021). Obviously, exogenous ions (Na^+^ or K^+^) would be introduced to the medium during this pH adjustment. Our study indicated that the negative effect of pH adjustment by using these alkali was not obvious during the batch cultivation phase (24 h) and even the early feeding phase before 72 h, but the growth of freshwater green alga *C. sorokiniana* CMBB276 was severely inhibited by the accumulated salt (NaCl) caused by continuous pH adjustment during long-time fed-batch cultivation (Figure 4). Obviously, the biomass concentration could not be improved by direct replacement of NaOH solution with ammonium hydroxide. Under this condition, the calculated final average C/N ratio was as low as 5.96 and the maximum biomass concentration was only 91.3 g l^−1^ due to extra introduction of nitrogen from ammonium hydroxide by pH adjustment (Table 1, Strategy 2#). Our results indicated that the biomass concentration could be further improved by reducing the N concentration of the feeding medium when ammonium hydroxide was used for pH maintenance, and an ultrahigh biomass concentration of 232 g l^−1^ was achieved by greatly reducing the N concentration of the feeding medium from C/N ratios 18 to 360 (Table 1).

Protein production from microalgae requires high cell density during cultivation, and heterotrophic microalgae can achieve high cell density and yet are confronted with the problem of low protein content (Xie et al., 2017). Although a relatively high biomass concentration (232 g l^−1^) and the highest protein yield (86.55 g l^−1^) were achieved in this study (Supplementary Table S1), the protein content of the high concentration *C. sorokiniana* CMBB276 cells (37.3% of DCW) is still much lower than that of other reported *Chlorella* species (Supplementary Table S1). Commercial *Chlorella* biomass products usually have protein content above 50% of DCW. Several methods were explored for enhancing the protein content, including sequential heterotrophic/autotrophic cultivation (Ogbonna et al., 1997), the utilization of plant growth substances (Bajguz and Piotrowska-Niczyporuk, 2014), and nitrogen concentration-shift cultivation (Xie et al., 2017). The protein content of *C. sorokiniana* CMBB276 under a final average C/N ratio of 5.93 reached as high as 53.4% of DCW (Table 1, Strategy 2#), indicating the potential of C/N ratio regulation on the improvement of *C. sorokiniana* CMBB276 protein content. A two-stage C/N ratio regulation strategy by shifting the nitrogen concentration of the feeding medium from a high C/N ratio to a low C/N ratio might further improve the protein content while maintaining a high biomass concentration, which will be investigated in our future study.

## Conclusion

An ultrahigh-density heterotrophic culture process for the high protein-yielding unicellular alga *C. sorokiniana* CMBB276 was established with a novel nitrogen supply strategy by simultaneously reducing the concentration of nitrogen in the feeding medium and adjusting culture pH with ammonium hydroxide, which provides a paradigm for the heterotrophic cultivation of other freshwater microalgae with low salt tolerance. The high-cell-density culture technology established in this study is promising for the cost-effective production of protein-yielding *Chlorella* biomass for food and feed applications.

## Data Availability

The original contributions presented in the study are included in the article/Supplementary Material, further inquiries can be directed to the corresponding author.
