# Supplementary material for: Heterotrophically Ultrahigh-Cell-Density Cultivation of a High Protein-Yielding Unicellular Alga Chlorella With a Novel Nitrogen-Supply Strategy
Source: Front Bioeng Biotechnol. 2021 Nov 22;9:774854. doi: 10.3389/fbioe.2021.774854 (PMC8646024; doi:10.3389/fbioe.2021.774854)
Supplement: Supplementary file 1 [file Table1.DOCX]

**Heterotrophically ultrahigh-cell-density cultivation of a high protein-yielding unicellular alga *Chlorella* with a novel nitrogen-supply strategy**

Quan Xu^a,f^, Guoli Hou^a^, Jianping Chen^a^, Hongxia Wang^a^, Li Yuan^a^, Danxiang Han^a,d,e^, Qiang Hu^a,b,c,d^, Hu Jin^a*^

^a^ Center for Microalgal Biotechnology and Biofuels, Institute of Hydrobiology, Chinese Academy of Sciences, Wuhan 430072, P. R. China

^b^ Institute for Advanced Study, Shenzhen University, Shenzhen 518060, P. R. China

^c^ State Key Laboratory of Freshwater Ecology and Biotechnology, Institute of Hydrobiology, Chinese Academy of Sciences, Wuhan 430072, P. R. China

^d^ Key Laboratory for Algal Biology, Institute of Hydrobiology, Chinese Academy of Sciences, Wuhan 430072, P. R. China

^e^ The Innovative Academy of Seed Design, Chinese Academy of Sciences, Beijing 100864, P. R. China

^f^ College of Advanced Agricultural Sciences, University of Chinese Academy of Sciences, Beijing 100049, P. R. China

*Corresponding author: Hu Jin (jinhu@ihb.ac.cn)

**Supplementary data**

**Table S1**. Summary of major fermentation performances of *Chlorella species* under heterotrophic cultivation

| Algal species | Maximum biomass conc. (g L^-1^) & protein content (%, DCW) | Maximum protein yield  (g L^-1^) | References |
| --- | --- | --- | --- |
| *Chlorella sorokiniana* | 9.5 (37.8) | 3.59 | Haske-Cornelius et al. (2020) |
| *Chlorella vulgaris* | 3.04 (53.8) | 1.64 | Li et al. (2012) |
| *Chlorella protothecoides* | 10.68 (47.11) | 5.03 | D’Este et al. (2017) |
| *Chlorella vulgaris* | 117.2 (37.5) | 43.95 | Doucha and Livansky (2012) |
| *Chlorella regularis* | 90 (63) | 56.7 | Sansawa and Endo (2004) |
| *Chlorella protothecoides* | 64 (34.52) | 21.76 | Ceron-Garcia et al. (2013) |
| *Chlorella protothecoides* | 91.4 (25) | 22.85 | Wang et al. (2016) |
| *Chlorella sorokiniana* GT-1 | 271 (27.62) | 74.85 | Jin et al. (2021) |
| *Chlorella sorokiniana* SLM2 | 93 (33) | 30.69 | Jin et al. (2021) |
| *Chlorella sorokiniana* CMBB276 | 232 (37.3) | 86.55 | This study |
| *Chlorella sorokiniana* CMBB276 | 91.3 (53.4) | 48.75 | This study |

**References**

Ceron-Garcia, M. C., Macias-Sanchez, M. D., Sanchez-Miron, A., Garcia-Camacho, F., and Molina-Grima, E. (2013). A process for biodiesel production involving the heterotrophic fermentation of *Chlorella protothecoides* with glycerol as the carbon source. *Appl. Energy* 103, 341-349. doi: 10.1016/j.apenergy.2012.09.054

D'Este, M., Alvarado-Morales, M., and Angelidaki, I. (2017). *Laminaria digitata* as potential carbon source in heterotrophic microalgae cultivation for the production of fish feed supplement. *Algal Res.* 26, 1-7. doi: 10.1016/j.algal.2017.06.025

Doucha, J., and Livansky, K. (2012). Production of high-density *Chlorella* culture grown in fermenters. *J. Appl. Phycol.* 24, 35-43. doi:10.1007/s10811-010-9643-2

Haske-Cornelius, O., Vu, T., Schmiedhofer, C., Vielnascher, R., Dielacher, M., Sachs, V., et al. (2020). Cultivation of heterotrophic algae on enzymatically hydrolyzed municipal food waste. *Algal Res.* 50:101993. doi: 10.1016/j.algal.2020.101993

Jin, H., Chuai, W., Li, K., Hou, G., Wu, M., Chen, J., et al. (2021). Ultrahigh-cell-density heterotrophic cultivation of the unicellular green alga *Chlorella sorokiniana* for biomass production. *Biotechnol. Bioeng.* doi: 10.1002/bit.27890.

Li X., Wang W., Zhang Y., Zhang P., Xie T., Liang B. (2012). Effect of nitrogen concentrations on growth and protein content of heterotrophic microalgae. *Science and Technology of Food Industry* 33, 222-224.

Sansawa, H., and Endo, H. (2004). Production of intracellular phytochemicals in *Chlorella* under heterotrophic conditions. *J. Biosci. Bioeng.* 98, 437–444. doi: 10.1263/jbb.98.437

Wang, T., Tian, X., Liu, T., Wang, Z., Guan, W., Guo, M., et al. (2016). Enhancement of lipid productivity with a novel two-stage heterotrophic fed-batch culture of *Chlorella protothecoides* and a trial of CO_2_ recycling by coupling with autotrophic process. *Biomass Bioenerg.* 95, 235-243. doi:10.1016/j.biombioe.2016.10.010
